# NavGraph: Enhancing Blind Travelers’ Navigation Experience and Safety

**DOI:** 10.1145/3749537

**Published:** 2025-09-03

**Authors:** SERGIO MASCETTI, DRAGAN AHMETOVIC, GABRIELE GALIMBERTI, JAMES M. COUGHLAN

**Affiliations:** University of Milan, IT; University of Milan, IT; University of Milan, IT; The Smith-Kettlewell Eye Research Institute, USA

**Keywords:** Visual impairments, Orientation & Mobility, Navigation assistance, **Human-centered computing** → *User studies*, Ubiquitous and mobile computing systems and tools, Social and professional topics → People with disabilities, Assistive technologies

## Abstract

Independent navigation remains a significant challenge for blind and low vision individuals, especially in unfamiliar environments. In this paper, we introduce the Parsimonious Instructions design principle, which aims to enhance navigation safety while minimizing the number of instructions delivered to the user. We demonstrate the application of this principle through NavGraph, a navigation application adopting a modular architecture comprising four components: localization, routing, guidance, and user interface. NavGraph is designed to provide effective, non-intrusive navigation assistance by optimizing route computation and instruction delivery. We evaluated NavGraph in a user study with 10 blind participants, comparing it to a baseline solution. Results show that NavGraph significantly reduces the number of instructions and improves clarity and safety, without compromising navigation time. These findings support the potential of the Parsimonious Instructions design principle in assistive navigation technologies.

## 1 INTRODUCTION

Independent navigation is a major challenge for blind and low vision (BLV) travelers. Standard Orientation and Mobility (O&M) techniques using the long cane or guide dog are effective in helping BLV travelers navigate safely [109], however, independent travel is often avoided [31] or limited to familiar routes such as work-home commute [56]. In unfamiliar environments, in particular, BLV travelers often require sighted assistance [110]. Navigation assistance can also be provided through accessible smartphone navigation apps such as Google Maps and Apple Maps [63] or from navigation applications specifically designed for BLV people like BlindSquare [20] or AriadneGPS [49].

A fundamental challenge in the design of a navigation system for BLV people is to balance two contrasting needs [73, 91]. On the one hand, the users need to receive timely and accurate instructions to ensure that they are safely following the intended route [98]. On the other hand, the users should not be overwhelmed by too many instructions that can also pose safety hazards since the users also need to pay attention to the dangers present in the environment [105].

The importance of safety for BLV navigators, especially those traveling independently, cannot be overstated. Indeed, it is one of the premises of Orientation & Mobility training [109], which is essential for enabling safe, independent travel. Traffic intersections are especially dangerous travel locations, with infrastructure such as accessible pedestrian signals [100], crosswalks [9] and curb cuts with detectable warning surfaces [53] developed to enhance safety for all. Safety is also a cornerstone of the design and proper use of BLV navigation aids [45, 68]. Moreover, with the widespread use of rideshare services and the advent of autonomous and semi-autonomous vehicles, issues related to maintaining situational awareness and user trust both inside and outside of vehicles are coming to the fore [22, 37, 83].

To address the above challenge, in this paper we formulate the *Parsimonious Instructions* design principle, which aims to reduce the number of instructions provided to the BLV user while, at the same time, guaranteeing navigation safety. We then show how the application of this design principle impacts the design of a novel navigation assistance system prototype, called **NavGraph**, in terms of the routing procedure and the selection of the instructions to convey to the user. **NavGraph** was evaluated in comparison to a baseline solution that adopts the same localization technique, route model, and user interface, but differs in the route computation and selection of the instructions. Experimental results, conducted with 10 BLV participants, show that **NavGraph** significantly increases the system safety (in terms of time spent in the areas where it is safe to walk) while also significantly reducing the number of instructions and making the system significantly clearer for the users.

To summarize, the paper makes the following contributions:

- We formulate the *Parsimonious Instructions* design principle.
- We show how the *Parsimonious Instructions* design principle impacts the design of a navigation system for BLV people.
- We implement the *Parsimonious Instructions* design principles inside **NavGraph**, a navigation assistance prototype.
- We experimentally evaluate **NavGraph** and compare it with a baseline solution, providing statistical evidence of the impact of the proposed solutions.

## 2 RELATED WORK

In this paper, we decompose the assistive navigation problem into four layers. This conceptual organization and its relationship with the previous literature are described in Section 2.1. Then, in the remainder of this section, we describe the related work by identifying, for each relevant paper in the literature, its main contribution(s) according to the four layers. We note that, while publications on navigation address various subsets of these four layers (for instance, [65] proposes a subdivision into the three layers of localization, routing and user interface), we are unaware of prior work in this domain that explicitly addresses all four layers. Finally, after describing related work on each of the four layers, in Subsections 2.6 and 2.7 we briefly review past work on designing safe navigation systems for BLV people and on alternative navigation approaches (alternative to the turn-by-turn paradigm) for which the decomposition into four layers does not apply.

### 2.1 Problem Decomposition

**Fig. 1.**
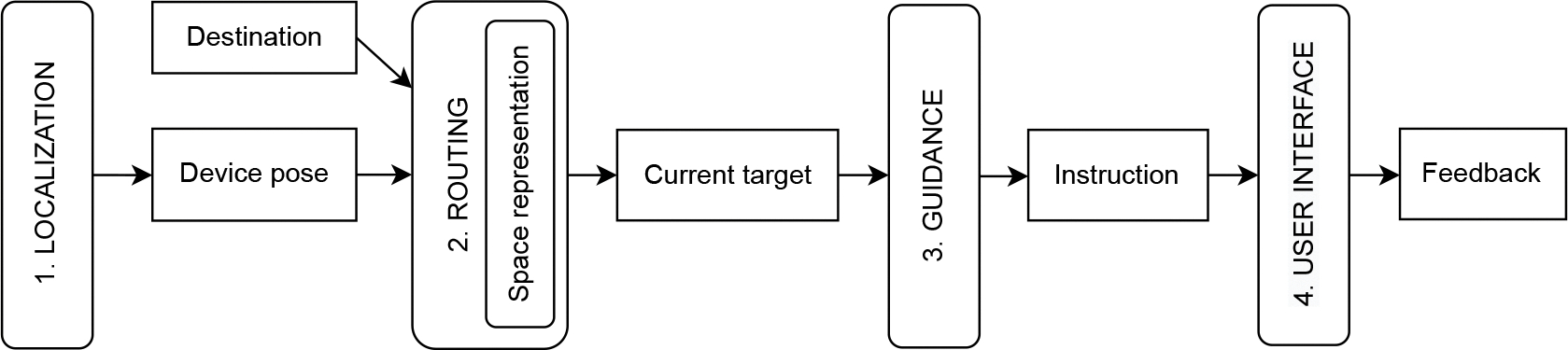
Decomposition of the navigation problem.

Kannan et al. suggested decomposing the navigation problem into three sub-problems: localization, routing and user interface [65]. In this paper we extend this logical organization to include the *guidance* layer, which addresses the problem of selecting the instructions to guide the user towards the destination. The resulting problem decomposition into four sub-problems is depicted in Fig. 1 and the layers are described in the following.

- *Localization* is the problem of computing the user’s pose, usually in terms of a 2D position and 1D orientation. Different technologies and techniques can be adopted to address this problem, either outdoor or indoor, including GNSS [66], Bluetooth beacons [7], or visual-inertial odometry [58].
- *Routing* is the problem of deciding where the user should be directed. Solving this problem generally involves computing a multi-step route from the current position to the destination. We do not address the problem of how the user selects the destination, because it depends on the specific domain. For example, in a road-navigation app, the user might type the address, while a museum application can present a set of artworks as possible destinations. Once a destination is selected, to compute the route, the *Routing* module uses a *space representation*: a set of data structures defining the relevant characteristics of the environment. We formalize the *space representation* used in our work in Section 3.1. The route computed by the *Routing* module is represented as a list of *intermediate targets* that the user should reach. The *current target* is the first element of this list and represents the point where the user should be directed. Once reached, the current target element is removed from the list, making the following element the new current target. As we motivate in this paper, instead of guiding the user directly towards the current target, in some cases it is convenient to guide the user towards a larger region that contains the current target and that we call the *current target area*.
- *Guidance* is the problem of selecting the instructions to guide the user toward the current target. The problem is particularly relevant for the navigation of BLV people because there is a need to provide salient instructions without overwhelming users with too much information (*e.g.*, audio feedback) [8, 85].
- *User interface* is the problem of conveying the guidance instructions to the users, using a combination of audio, haptic, and visual information. In particular, for audio information, it is possible to use combinations of synthesized speech, sonification [55], and earcons [19].

### 2.2 The *Localization* Problem

While in this paper we do not address the problem of how to accurately compute the user’s position, many prior works in the field of assistive navigation for BLV people address this topic. This is because assistive technologies supporting the mobility of BLV people require high localization accuracy [7, 24, 47].

Several outdoor navigation systems use the Global Navigation Satellite System^1^ to compute location everywhere on Earth. Among others, commercial applications like Google Maps [80], Apple Maps [79], GoodMaps [48] and BlindSquare [20], and research applications like Navig [66] adopt this solution.

Radio-signal-based solutions have been adopted for indoor positioning. Wireless localization [1, 57, 93] uses the availability of wireless networks to track the position of a device in an environment. Common techniques used for wireless localization use Radio Frequency Identification (RFID) tags [26] or Bluetooth Low Energy (BLE) beacons [7, 71, 86]. To increase the accuracy, the techniques can also adopt a fingerprinting-based approach [7, 86, 97]. Also, to address problems related to the maintenance of positioning systems based on BLE, crowdsourcing solutions were proposed [46].

Inertial odometry [39, 95, 105] uses the data from device sensors such as magnetometer, accelerometer, and gyroscope to compute how the pose changes in time. The accuracy of this family of techniques can be increased by also considering visual features captured by the camera (visual-inertial odometry) [13, 29, 42, 66, 114]. Since odometry and visual-inertial odometry are subject to drift problems (position accuracy decreases over time) they are also used in combination with other techniques that provide sporadic position fixes [30, 42]. These techniques can also be conveniently implemented by relying on existing augmented reality libraries [2, 5, 27, 114] and computer vision algorithms [51, 60, 61, 88].

Regardless of the technical approach used, many solutions also adopt Particle Filtering techniques to improve localization accuracy [43, 52, 87, 95]. Particle Filtering techniques are sequential probabilistic methods used for estimating the state of a system by representing the state distribution with a set of particles, which are updated based on observations and resampled to improve accuracy. These solutions also model the environment constraints (*e.g.*, areas where the user is not expected to go) to achieve more robust localization [42, 87, 95].

### 2.3 The *Routing* Problem

Many existing routing solutions adopt a turn-by-turn navigation paradigm, hence modeling the space with a graph in which the nodes represent the turning points and the edges represent the straight paths along which the user is expected to walk (*e.g.*, an aisle) [2, 7, 12, 25, 61, 97]. A graph-based space modeling makes it possible to compute the shortest route between any pairs of nodes on the graph by weighting each edge with the geographical distance between its two nodes. Different edge weights can model different interpretations of *best* route, for example, to balance distance with other factors, such as possible hazards along the route. The routing solution proposed in this paper does not focus on how to compute the weights for obtaining the shortest path. Existing approaches focusing on this aspect can be integrated in our technique, in particular in the short route computation mentioned in Line 10 of Algorithm 1. An alternative space representation was proposed by Ren et al. [95] and is based on a discretization of the space into *tiles* (*i.e.*, non-overlapping polygons).

Although route computation is a well-known problem in generic navigation applications, there are specific problems that are particularly relevant to navigation assistance for people with visual impairments, which we address in this paper.

- **Definition of navigation areas**. In some contexts, it is necessary to model the fact that the user should not or cannot move too far away from the edge (*e.g.*, if the edge represents a sidewalk). One possible solution briefly sketched in a previous paper is to define an area around each edge where the user can freely move [2]. In this paper we extend this idea by proposing the concept of *navigation area* (see Section 3.1).
- **Target updating**. In turn-by-turn navigation, the user is directed along a straight line to reach a turning point, and, once it is reached, the user is directed toward the following one. Previous solutions update the target when the user is closer than a given threshold to the turning point [2, 7, 95]. Instead, in this paper we present a routing procedure that seamlessly addresses the target updating problem by relying on the concept of navigation areas (see Section 3.2).

### 2.4 The *Guidance* Problem

Most existing solutions do not report an explicit and complete procedure that defines how to generate navigation instructions. Some papers briefly mention the problem, for example, reporting that a rotation instruction is generated when there is a need to correct the user’s orientation [2–4, 7]. Other papers report the need to inform the user that the rotation was completed [2–4, 7, 8] and focus on the reaction time to navigation instructions [10, 89]. In contrast, in this paper we specifically focus on the problem of selecting which instructions to provide and we show that the *Guidance* module (Section 3.3) has a significant impact on safety and on user experience.

### 2.5 The *User Interface* Problem

For the *User Interface* problem, there are several solutions to convey information to a BLV traveler. In particular, audio feedback-based interfaces are widely explored in research. In this domain, previous papers have adopted various solutions, including spoken instructions [12, 14, 95], sonification techniques [2, 3, 23, 70, 92], earcons [4, 66], and their combination [4, 21, 36]. Audio feedback may be used to convey both the distance and/or direction to the target. For example, Fiannaca et al. [36] propose Headlock, which provides audio feedback (sonification and speech) to guide a user across an open space to a doorway. The NavCog system [7, 97] uses a combination of spoken instructions combined with earcons and sonifications to provide guidance to a destination. For a straight path, the user is periodically informed about the distance to the current target with synthesized verbal messages. For turns, once the user is aligned correctly to the new required direction (based on gyroscope data), a confirmation sound is played. Different types of sonification have been studied in the literature to better support the user during rotations (which are particularly critical) [3, 4] and have also been adapted to guide users along straight paths [2] and to avoid obstacles [92].

Earcons and auditory icons are used to alert the BLV user to specific situations. Cosantinescu et al. [28] used auditory icons to provide an awareness of the scene framed by a head-mounted camera. Participants in their study preferred an interface using these auditory icons over speech-based earcons (spearcons), which may be difficult to understand if delivered rapidly.

Haptic feedback is another modality that can be used to convey navigation instructions [16, 38, 64, 67, 69, 99]. Different types of hardware devices were experimented with in the literature to convey the navigation instructions with tactile feedback, including vibrotactile bracelets [64, 99], haptic strap [67], head-mounted haptic sensors [78, 113], haptic cues underfoot [112], natural-sound-like vibrations [69], smartphone vibration [16] and belts [38]. The combination of haptic feedback with audio feedback is also explored in research [75, 116].

Navigation information can also be provided graphically, to support the navigation of people with low vision. This approach was explored by Zhao et al. [115] who compared audio and visual feedback concluding that visual feedback can provide lower cognitive load during navigation for people with low vision.

This paper does not focus on the *User Interface* sub-problem and adopts a previous solution [3] based on a combination of vocal instructions, earcons and sonification.

### 2.6 Providing Accurate Guidance Instructions Without Overwhelming the User

Several papers in the state of the art discuss the problem of providing accurate guidance without overwhelming the user with too many audio instructions. The work of Tsai et al. discusses the need for the navigation user interface to pose minimal disruption to the user, who must concentrate while walking in an unfamiliar environment [105]. Similarly, Sato et al. address the extra cognitive load imposed on a navigation app user by the addition of a user interface that reports semantic features in the environment, and how this load impacts safety [98]. The work of Panëels et al. explores the tradeoffs between quality versus quantity in audio feedback for a BLV navigation system, and also investigates the use of spatialized sound as an intuitive interface for exploring nearby points of interest [91]. The specific needs of, and strategies adopted by, BLV travelers at traffic intersections [108] and work zones [106] should also be considered in the design of navigation systems. In addition, ambient noise can make it difficult for users to understand synthesized speech, which is a key user interface modality for BLV individuals provided by screen readers, motivating the need to develop techniques to enhance screen reader intelligibility [6]. This paper builds upon current state-of-the-art results and addresses the challenge of reducing the amount of audio information while preserving a system’s ability to accurately and safely guide the user.

### 2.7 Alternative Navigation Approaches

**NavGraph** adopts a turn-by-turn paradigm because it is common in accessible navigation solutions [2, 7, 12, 25, 61, 97]. However, alternative approaches exist, notably the use of spatialized interfaces that indicate a desired direction of travel towards a destination using spatial rather than verbal or semantic feedback [62]. One form of this spatialized interface guides the user to aim their smartphone or other (typically handheld) device towards the target direction. This is accomplished by continuously modulating auditory and/or haptic feedback to signal the discrepancy between the current pointing azimuth (direction along the horizon) and the target azimuth [29, 45, 95]. An alternative spatialized interface uses 3D (spatialized) audio to indicate a target direction. In this approach [76, 103, 104], the target direction is represented by a virtual beacon or other sound emanating from a fixed direction in the environment (the binaural sound must be continuously updated to compensate for the user’s head rotations); using binaural audio cues, the user can estimate the beacon direction and rotate their head towards it. While this approach requires special hardware (such as Apple AirPods) that support binaural audio and head azimuth tracking, it is intuitive and typically imposes a lower cognitive load than verbal instructions [72].

## 3 SOLUTION DESIGN

To address the problem of overwhelming the user with too many audio instructions, we seek to minimize the number of audio instructions (in particular route corrections) issued to the user, while preserving the user’s safety. To this aim, we formulate the following *Parsimonious Instructions* design principle.

***Parsimonious Instructions* design principle** The navigation system should safely guide the user toward the destination by using as few audio instructions as possible. This should be achieved by providing audio instructions only when needed and by preventing situations in which additional audio instructions are likely required.

In the following of this section, we show how the *Parsimonious Instructions* principle impacts the routing and guidance subproblems by presenting two modules addressing these subproblems (see Sections 3.2 and 3.3, respectively). To clearly present the suggested routing procedure, we first formalize the space representation in Section 3.1.

### 3.1 Space Representation

In this paper, we model the user position over a two-dimensional space S. Even though the user’s and device’s pose do not necessarily match (in particular for the orientation) in the following, for the sake of simplicity, we refer to the *user*’s position u.p∈S and orientation u.o∈[0,2π).

We define a *Navigation Graph* where edges and nodes represent straight paths and turning points, respectively. The *Navigation Graph* is used to compute the routes and hence a weighting function among edges is required. Different weighting functions can be defined, for example taking into account the expected effort to traverse an edge [35, 40]. Formally, the *Navigation Graph* 𝒢 is an undirected and weighted graph ⟨N,E,W⟩ where N is a set of nodes, $E\subseteq N^{2}$ a set of edges, and W a weighting function $W:E\to R^{+}$. We denote the position of each node n in S as n.p An example of a weighting function that only takes into account the length of an edge in S can be defined as follows: for each $e=\left\langle n_{1},n_{2}\right\rangle \in E,\;w(e)=dist\left(n_{1}.p,n_{2}.p\right)$, where dist is the Euclidean distance.

**Fig. 2.**
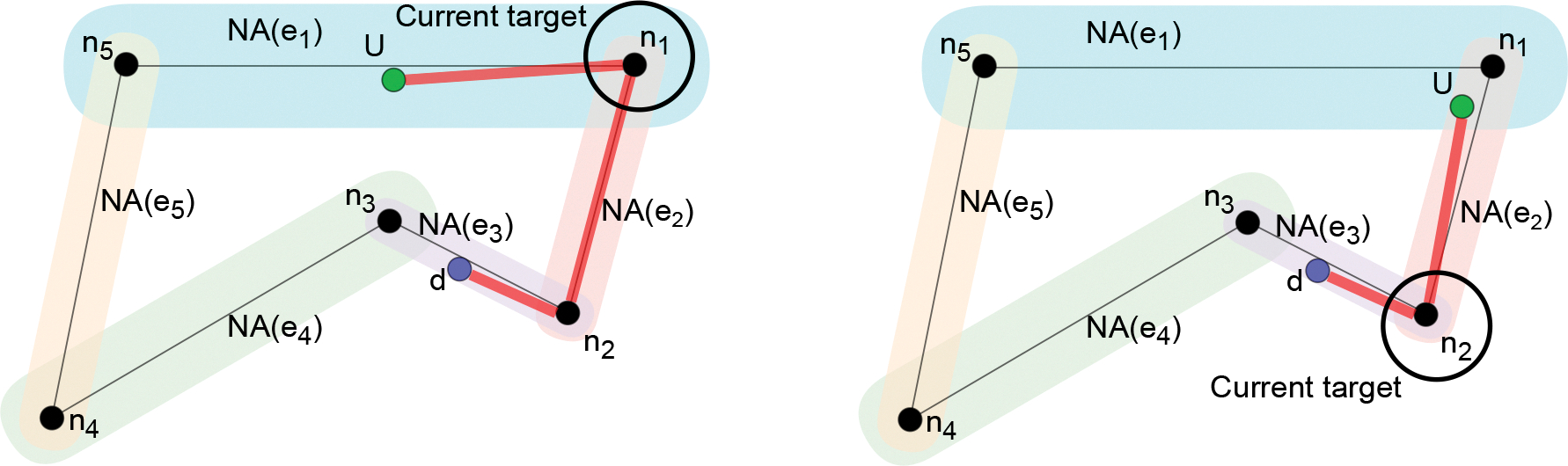
Example of the space representation and of the routing from the user’s position (U) to the destination position (d).

Each edge intuitively defines a straight path that the user can follow. In some cases, the users are constrained to walk along an edge (*e.g.*, in a corridor), while in open spaces users can walk freely, possibly moving away from the edge they should be following. To represent this, we introduce the concept of *Navigation Area* of an edge, which is the region of space around that edge. The union of all Navigation Areas for all edges in the graph is the *Graph Navigation Area*. Formally, for each edge e, we define a linear distance $\delta_{e}$ and we define the navigation area of e as the set of points closer than $\delta_{e}$ to e:

$$NA(e)=\left\{p\in S|\mathrm{dist}(p,e)<\delta_{e}\right\}$$

where, given $e=\left\langle n_{1},n_{2}\right\rangle ,\;\mathrm{dist}(p,e)$ is the euclidean distance between p and the line segment $\bar{n_{1}n_{2}}$. Fig. 2a shows an example of a *Navigation Graph* with five edges, each with its navigation area.

We model the destination d as a point in S. We assume that the user has reached the destination if the user is in the *destination area*: a circle centered in the destination with radius $\delta_{d}$. Since the Graph Navigation Area defines where the user is expected to walk, we assume that the system should prevent the user from moving outside it. For this reason, we assume that the destination area is contained in the Graph Navigation Area.

### 3.2 The *Routing* Module

The objective of the *Routing* module is to process the space representation, taking into account the user’s position and the destination to compute intermediate targets and, in particular, the current target, which is then provided to the guidance module. Considering the space representation formalized in Section 3.1, there are the following four cases.

1. The user has arrived at the destination *i.e.*, the user position is in the destination area.
2. The user and the destination are in the navigation area of the same edge (*i.e.*, ∃e∈E∣u.p∈NA(e)∧d∈NA(e). In this case the user can be directly guided toward the destination.
3. The user is outside the graph navigation area and should be guided to re-enter in the navigation area.
4. In all other cases, the *Routing* module computes the route from the user’s position to the destination along the graph navigation area. The remainder of this section describes this procedure.

#### Intuition.

Let’s first consider the case in which the user’s position and the destination are each contained in the navigation area of a single edge. An example is shown in Fig. 2a. In this case, the user is in the navigation area of edge $e_{1}$ and can be guided toward $n_{1}$ or $n_{5}$ along the navigation area. Then, from either $n_{1}$ or $n_{5}$ the user can follow the *Navigation Graph* toward $n_{2}$ or $n_{3}$, which are the vertexes of $e_{3}$. From there, the user can be guided toward the destination along the navigation area of $e_{3}$. In a more general case, the user position and/or the destination can be in the navigation area of more than one edge. The example in Fig. 2b shows the case in which the user’s position is contained in the navigation areas of two edges (i.e., $e_{1}$ and $e_{2}$) and hence the user can be guided toward any node among $n_{1},\;n_{2}$, and $n_{5}$.

#### Computation.

Algorithm 1 shows the procedure to compute the route. It takes as input the user’s position, the destination and the *Navigation Graph*. The procedure first creates two new nodes, $n_{u}$ and $n_{d}$, representing the user’s position and the destination, respectively (Lines 1 and 2). The procedure then computes the set $NA_{u}$ of edges whose navigation area contains the user position and, similarly, the set $NA_{d}$ of edges whose navigation area contains the destination (Lines 3 and 4). In Line 5 the procedure computes the set of nodes $N_{u}$ that are the vertexes of edges in $E_{u}$. Similarly (Line 6) it computes the set $E_{d}$ for the destination. Next, the procedure creates the set of edges $E_{u}$ that contains, for each node n in $N_{u}$ one edge between $n_{u}$ and n. Similarly for the destination (Lines 7 and 8). Then, the procedure extends 𝒢 by adding $n_{u}$ and $n_{d}$ to the set of nodes and $E_{u}$ and $E_{d}$ to the set of edges. Finally, the procedure returns the shortest route on ${𝒢}^{\prime }$ between $n_{u}$ and $n_{d}$.

**Algorithm 1:** Routing procedure

**Input:** u.p,d,𝒢=⟨N,E,W⟩	
**Output:** shortest route from u.p to d along the navigation area of 𝒢.	
1:	$n_{u}$ is a node representing the user’s position
2:	$n_{d}$ is a node representing the destination position
3:	$NA_{u}=\{e\in E|u.p\in NA(e)\}$
4:	$NA_{d}=\{e\in E|d\in NA(e)\}$
5:	$N_{u}={⋃}_{e=\left\langle n_{1},n_{2}\right\rangle \in NA_{u}}\{n1,n2\}$
6:	$N_{d}={⋃}_{e=\left\langle n_{1},n_{2}\right\rangle \in NA_{d}}\{n1,n2\}$
7:	$E_{u}={⋃}_{n\in N_{u}}\left\langle n_{u},n\right\rangle$
8:	$E_{d}={⋃}_{n\in N_{d}}\left\langle n_{d},n\right\rangle$
9:	${𝒢}^{\prime }=\left\langle N\cup n_{u}\cup n_{d},E\cup E_{u},\cup E_{d},W\right\rangle$
10:	**return** the shortest route on ${𝒢}^{\prime }$ between $n_{u}$ and $n_{d}$

*Application of the* Parsimonious Instructions *Design Principle.* The proposed routing solution adopts the *Parsimonious Instructions* principle since it does not require the user to arrive in close proximity of the current target, an action that can require additional instructions to complete. We call this the **current target update** problem which consists in deciding when to pop the current target from the list of intermediate targets, thus directing the user to the next target in the list. The proposed routing technique updates the current target when the user enters in the navigation area of a new edge. Consider for example the situation in Fig. 2: the user is initially guided towards $n_{1}$ (Section 3.3.2 describes how this is achieved). Before arriving at $n_{1}$, the user enters in the navigation area of $e_{2}$ (see Fig. 2b). From this position, the proposed routing algorithm computes $n_{2}$ as the new current target, hence the navigation system can avoid guiding the user to $n_{1}$, ultimately reducing the number of messages.

### 3.3 The *Guidance* Module

In line with the previous literature [2, 97] the *Guidance* module generates four types of instructions: **arrived**, **walk**, **rotate**, and **side-step**. Considering the four cases described in Section 3.2, the *Guidance* module returns **arrived**, if the user arrived at destination (case 1). Instead, if the user is outside the graph navigation area (case 2), the aim is to guide them to return to the navigation area with **walk**, **rotate**, and **side-step**, as discussed in Section 3.3.1. In cases 3 and 4, the *Guidance* module guides the user towards a target (the destination or the current target, respectively) by using **walk** and **rotate** messages. This is discussed in Section 3.3.2.

#### 3.3.1 Guidance to the Navigation Area.

**Fig. 3.**
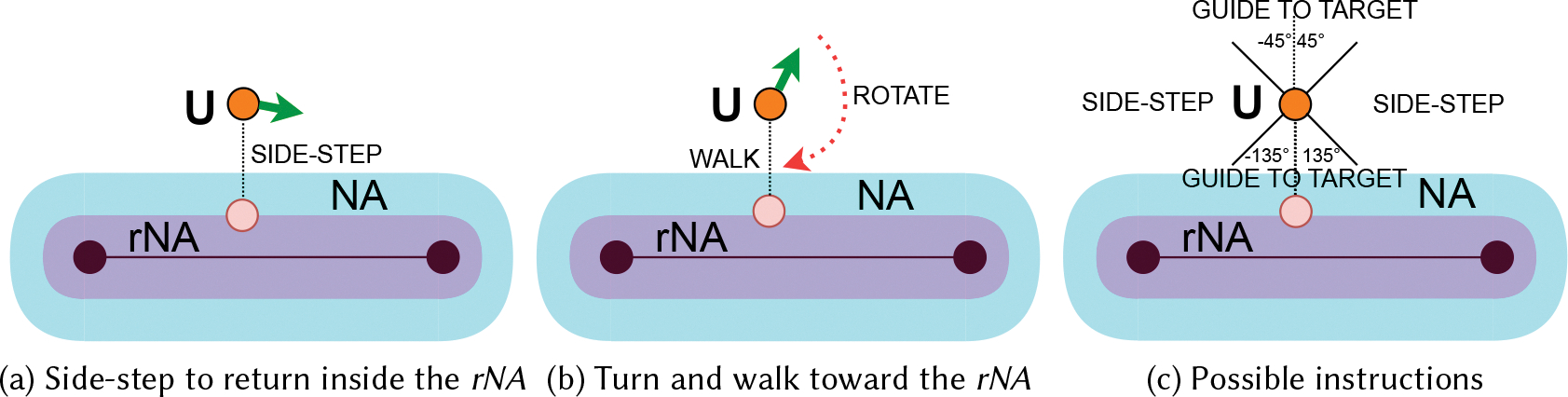
Instructions provided when the user U is outside the navigation area. The green arrow is the user’s orientation.

A common situation, during navigation along a straight path, is that the user veers, thus walking outside the navigation area. When this happens, it is common that the user is directed approximately in the correct direction (*i.e.*, parallel to the edge). Fig. 3a shows an example. In this case, it is possible to guide the user to re-enter into the navigation area with a **side-step** instruction (*e.g.*, a side-step toward the right, as in the example of Fig. 3a).

The *Guidance* module applies the *parsimonious correction* principle: instead of guiding the user to just re-enter into the navigation area, it guides the user deeper into the navigation area. The reason is that, if the user is guided to just re-enter the navigation area, there is the risk that the user could exit again from it after a short while. This problem is mitigated by guiding the user closer to the actual edge. Technically, we define a *restricted Navigation Area* (*rNA*), which has the same definition as the navigation area, but with a smaller $\delta_{e}$ (in our experiments we used a value for $\delta_{e}$ of the rNA that is half that of the navigation area). Once the user re-enters the *rNA*, guidance resumes as described in Section 3.3.2.

While the situation depicted in Fig. 3a is common, there are situations in which the *side-step* instruction would not guide the user back to the navigation area. This happens when the user orientation is almost perpendicular to the navigation area, as exemplified by Fig. 3b. In this case, there is the need to guide the user to **rotate** and then to **walk** to re-enter into the *rNA*. In this case, the *rNA* is defined as the current target area and the user is guided towards it as described in Section 3.3.2.

In order to decide whether to use a **side-step** instruction or define a current target area, we define an angular reference system oriented perpendicularly to the navigation area as depicted in Fig. 3c. If the user orientation is between 45° and 135°, the user is instructed to do a **side-step** to the right, otherwise, if the user orientation is between −135° and −45°, the user is instructed to do a **side-step** to the left. If, at any point while re-entering into the navigation area, the user orientation is not in one of these two ranges, the *rNA* is defined as the current target area and the user is guided with a sequence or **rotate** and **walk** messages as described in Section 3.3.2.

#### 3.3.2 Guidance to the Target.

**Fig. 4.**
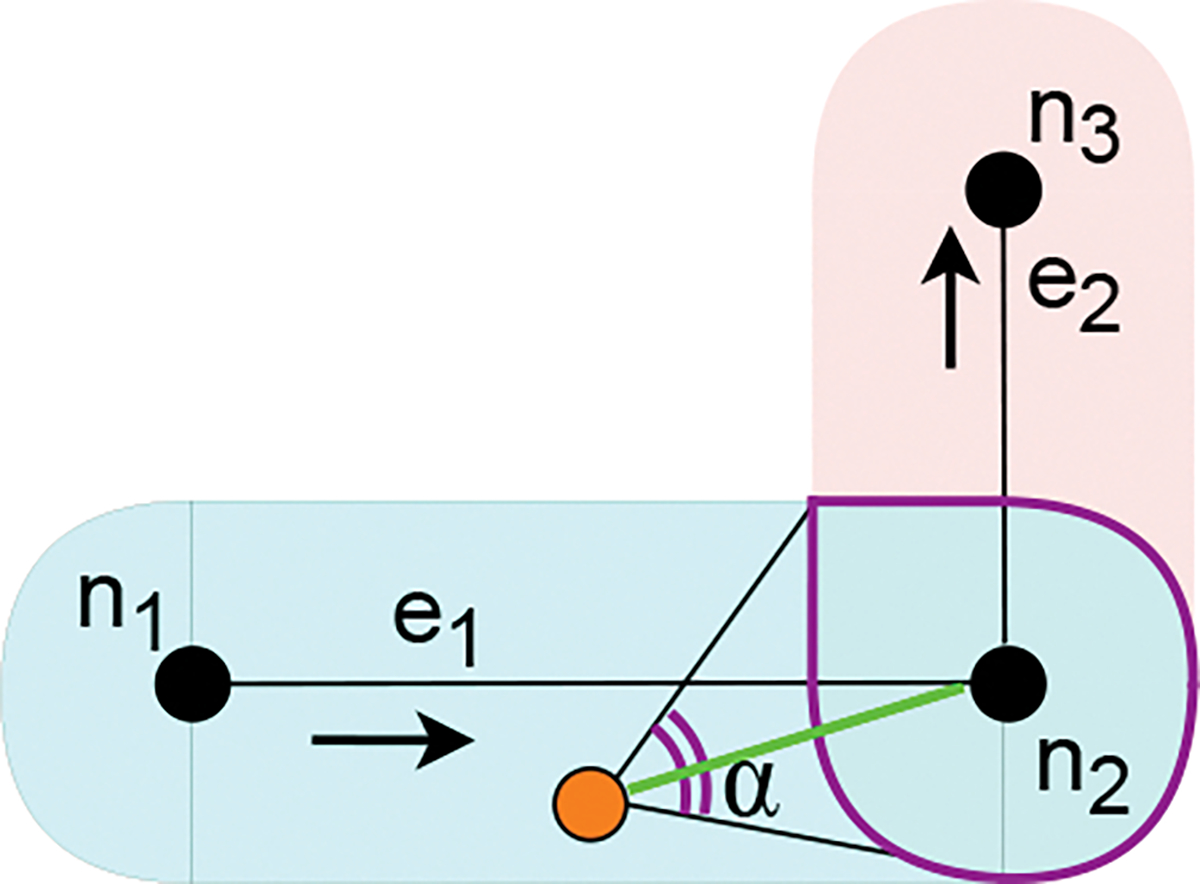
Example of current target area computed as the intersection of two navigation areas.

In the following we describe how the *Guidance* module creates instructions to guide a user towards the current target area. There are three situations in which the user can be guided towards the current target area:

- When the user is re-entering into the *rNA*. In this case the current target area is the *rNA* of the current edge (the edge along which the user was walking) as explained in Section 3.3.1.
- When the user and the destination are in the navigation area of the same edge (case 3 in Section 3.2). In this case, the current target area is the destination area.
- When the *Routing* module computes a route towards the destination (case 4 in Section 3.2). In this case, the current target area is the intersection between the navigation area of the current edge and the navigation area of the next edge (the edge defined by the first two nodes in the list of intermediate targets). We explain the intuition with the example of Fig. 4: the user is navigating along the current edge $e_{1}$ toward the intersection of the navigation areas of $e_{1}$ and $e_{2}$ (the area with purple outline). As soon as the user reaches this area, since the user is now in the navigation area of $e_{2}$, the routing procedure (Algorithm 1) computes a new shortest path having $n_{2}$ as the first node and hence the user will be guided towards the following current target area.

Guidance towards the current target area works as follows: if the user is heading towards the current target area, then a **walk** instruction is returned. Otherwise, the user is instructed to rotate to correct the orientation. This is formalized as follows: consider the angle α centered on the user’s position (see Fig. 4) subtending the current target area. As long as the user orientation is in α, then a **walk** instruction is returned. Otherwise, the *Guidance* module starts correcting the user’s orientation with a **rotate** instruction until the user is aligned with the bisector of α. Then, a **walk** instruction is returned again.

The procedure adopts the *Parsimonious Instructions* principle with two policies. First, the target toward which the user is directed is as large as possible. Consider the example in Fig. 4: instead of guiding the user toward $n_{2}$ (or a small area centered on $n_{2}$), which would likely require several instructions to keep the proper alignment, the *Guidance* module selects the intersection between $NA\left(e_{1}\right)$ and $NA\left(e_{2}\right)$ as the destination toward which the user should be directed. Clearly, the angle centered in the user’s position that subtends this area is larger, hence making it easier for the user to keep the right alignment, without the need for instructions to correct the orientation. Second, when the user direction is corrected, instead of performing the minimum orientation correction, which would direct the user to the edge of the current target area, the module requires the user to completely re-align to the center of the current target area.

Note that the above approach is subject to a problem: when the user is far away from the target (*e.g.*, on a very long edge), it could happen that α is a small angle, making it hard for the user to keep the orientation within this angle, hence resulting in frequent orientation corrections. For this reason, the size of α is computed as the maximum between the angle subtending the current target area and a fixed angular threshold (30° in our experiments).

## 4 USER STUDY

We conducted a user study with ten fully blind participants (see Section 4.5) to understand how the proposed architecture affects the navigation experience of people who are BLV. The study compared two applications: **NavGraph**, implementing the proposed approach, and a state-of-the-art (**SOTA**) solution (see Section 4.2) based on a prior work [2]. The study was approved by the ethics committee of the University of Milan, approval: 3/23^2^.

### 4.1 Experimental Design

The experimental evaluation followed a within-group design in which the primary independent factor (condition) was the navigation application used (**NavGraph** or **SOTA**). For a given condition, each participant did two navigation tasks (in total four different paths). Both the paths and the condition were alternated and counterbalanced across the experiments to avoid effects of order (see Section 4.4).

#### 4.1.1 Objective Metrics.

**Fig. 5.**
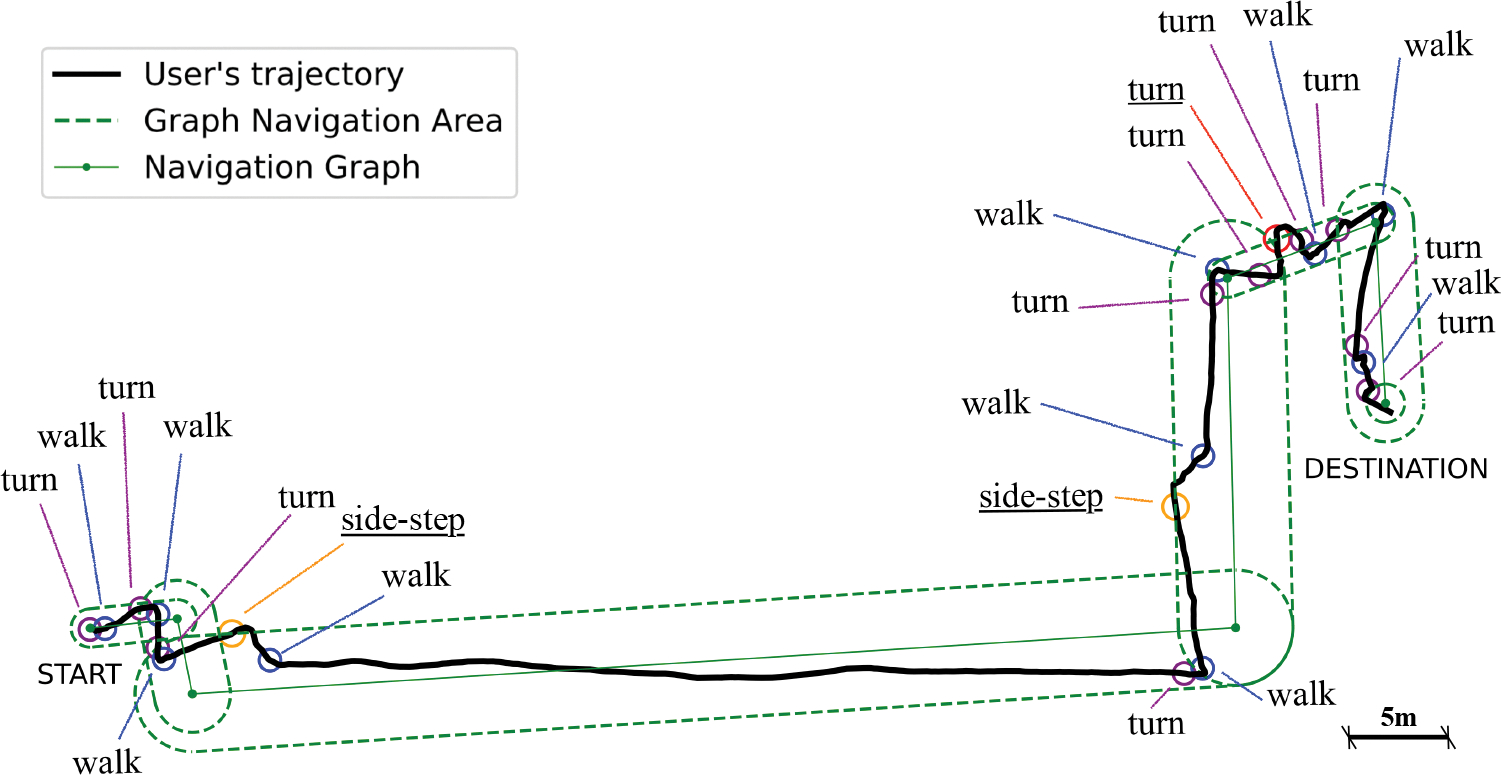
Example of a user’s trajectory with the instructions provided during the navigation. Underlined instructions are issued when the user is outside the navigation area.

Objective metrics were computed from the system logs, which included the user’s trajectory, navigation state, and the provided instructions. For example, Fig. 5 shows the data collected for the trajectory of **P2** on path 2. The green lines represent the *Navigation Graph* (solid line) and the navigation area (dashed lines). The user’s trajectory is represented in black. The figure also reports the instructions provided to the user during the navigation. Specifically, the following objective metrics were collected:

- **Number of instructions** was the main study metric, and it was subdivided by the type of the instruction as secondary factor (turn, walk, side-step);
- **Time duration** of the navigation task;
- **Time outside the navigation area**, subdivided by the width of the *Navigation Area* of the edge as secondary factor. This factor had three possible values ($\delta_{e}=\{1m,2m,3m\}$);
- **Lateral distance** from the current edge;
- **Instructions cluster**. Given a temporal parameter t, an instruction cluster is a set of consecutive instructions such that the temporal distance between each of them and the following is less then t. In this case the temporal between the clusters (t={0.5s,1s,2s}) was considered as secondary factor.

#### 4.1.2 Subjective Metrics.

Participants’ subjective feedback was collected through two types of questionnaires, one administered after each task, and one administered at the end of the experiment^3^. The questionnaire administered after each task consists of three Likert-like scale items, with answers ranging from 1 (disagree) to 5 (agree), and one open question. The supervisor also invited the participant to motivate or discuss their answers. In the following, we refer to each topic using the term reported in bold (*i.e.*, **Timeliness**, **Clarity**, **Conciseness**, **Completeness**).

- (Likert-like scale item.) The instructions were provided timely. (**Timeliness**)
- (Likert-like scale item.) The instructions seem clear to me. (**Clarity**)
- (Likert-like scale item.) The instructions are concise. (**Conciseness**)
- (Open question) Are the instructions complete? (**Completeness**)

At the end of all the tasks, we collected additional comments based on the following questions. Again, we refer to each topic using the term reported in bold (*i.e.*, **General differences** and **Quantity of instructions**).

- Did you perceive any differences between the two navigation apps? (**General differences**)
- Did you notice any differences in the quantity and frequency of instructions provided by the two navigation apps? (**Quantity of instructions**)

#### 4.1.3 Statistical Analysis.

Since we adopted a within-group design, with a primary independent factor (condition) and a possible metric-dependent secondary factor (see Section 4.1.1), we employed repeated measures factorial Analysis of Variance (ANOVA) as hypothesis test of choice [44]. When the data was not normally distributed, we used Aligned Rank Transform (ART), which allows to apply factorial ANOVA non-parametrically [111]. Normality of the distributions was checked using Shapiro-Wilk’s test [94]. Post hoc analyses were conducted using the Paired T-test and Wilcoxon signed-rank test for ANOVA and ART, respectively. When multiple comparisons were conducted, results were adjusted using the Benjamini-Hochberg False Discovery Rate method [17].

#### 4.1.4 Power Analysis.

To ensure that the results are statistically meaningful, we have conducted a power analysis after the first 8 experiments, with the goal of assessing the minimum sample size required for the study. For this purpose, we have used G*Power software [34] version 3.1.9.7. Results indicated that 10 participants were needed to reach the desired statistical power. We have therefore completed two additional experiments, reaching a total of 10 participants.

### 4.2 Experimental Apparatus

The study was conducted using an iPhone 14 Pro, running the two navigation applications: **NavGraph**^4^ and **SOTA**^5^. As **SOTA**, we implemented a baseline application instead of using an existing commercial software because we wanted the two solutions to differ only in the aspects related to the *Parsimonious Instructions* design principle (*i.e.*, *Routing* and *Guidance* modules). Indeed, any additional difference (*e.g.*, in the localization technique or in the user interaction) could be a confounding factor during the evaluation. Furthermore, the quantitative analysis reported in this paper relies on the acquisition of data logs generated with ad-hoc code. Thus, comparing with a commercial system would require to have access to its source code.

In the following, we describe the implementation of the four modules in the two applications. The differences between the two applications are summarized in Table 1. A video, provided in the supplementary material, shows examples of the two applications.

#### Localization.

The *Localization* module in both apps is implemented with a combination of visual-inertial odometry and image tracking enabled by native Augmented Reality libraries^6^. This solution was used as it is easy to implement and can achieve accurate and stable localization (and direction) if visual markers are placed at short intervals. Indeed, in our experiments, visual markers are placed in known locations (approximately every 5m) along the path, so that the drift due to inertial odometry is corrected during navigation.

#### Routing.

Both **NavGraph** and **SOTA** use the space representation described in Section 3.1 but they use a different routing procedure. **NavGraph** adopts the routing procedure described in Section 3.2, while the routing procedure of **SOTA** works as follows: when the navigation begins, **SOTA** computes the current target with the same procedure used in **NavGraph** (Algorithm 1). Then **SOTA** guides the user towards it. When the user’s distance from the next target is less than the $\delta_{e}$ value of the next edge, the current target is popped from the list of intermediate targets and the user is directed toward the following target in the list.

#### Guidance.

Both applications use the *Guidance* module presented in Section 3.3 except for two differences. First, when the user exists from the navigation area, **SOTA** issues a side-step, without guiding the user back to the restricted navigation area as described in Section 3.3. Second, **SOTA** issues a rotation instruction when the user is misaligned with respect to the current target by more than 30 degrees.

#### User Interface.

The *User Interface* module used for both **NavGraph** and **SOTA** is based on prior work [2], and implements a combination of verbal messages (rotate, walk, side-step), earcons and sonification, used to convey quantities (*e.g.*, the amount of rotation). The only difference between the two applications is a consequence of the differences in the *Guidance* module: in **SOTA** the user is only instructed to make a single lateral step (*e.g.*, “Step right”) while in **NavGraph** this is also followed by a sonification to guide the user to re-enter in the restricted navigation area.

To ensure that the device camera can correctly frame the visual markers, maintain a clear view of the surrounding environment, and have a consistent orientation in the direction of the participant’s body, the device was positioned on the participant’s chest, placed on a chest harness. The auditory information was provided through the device speaker, without using headphones, in order not to mask the surrounding soundscape, which is important for BLV travelers to avoid hazards.

### 4.3 Experimental Setting

The study was conducted on the campus of the University of Milan. We defined four routes, each with the same number of edges and turns, and approximately the same length (103m, 99m, 97m, and 101m, respectively). For each route, the turns were equally divided into left and right turns. Also, each route had two longer edges, two medium-length edges and two shorter edges, with navigation area width $\delta_{e}$ set to 3m, 2m and 1m, respectively. The routes were chosen to avoid hazards and objects that the participant can use to support orientation and navigation (like walls) because we wanted the participants to rely on the provided instructions only, without being guided by other objects in the environment. For example, we wanted to avoid having participants walk along a straight path by following a wall (BLV individuals are usually trained to walk along walls using the white cane or echolocation).

### 4.4 Experimental Protocol

The study was organized into four main phases: a preliminary questionnaire, study introduction, the navigation phase, and a final questionnaire.

The preliminary questionnaire collected the participants’ demographic data, expertise, and habits. The main results of the preliminary questionnaire are reported in Section 4.5.

In the second phase we briefly introduced the experiment, presenting an overview of the navigation tasks, and we explained to the participant how to follow the instructions provided by the application.

During the navigation phase, each participant was asked to complete four tasks. In each task, the participant would traverse one of the routes with one of the two apps. The app used and routes were counterbalanced using a Latin Square Design to account for the effects of order. Before each task, participants were introduced to the app used in the task through a short tutorial. In the tutorial the supervisor explained which instructions and sonifications are provided by the app and how to react to them. The participant was then asked to try the navigation system on a short test route consisting of two short straight paths and one rotation. Once the tutorial was completed, participants were accompanied by the supervisor at the starting point of the route and were asked to follow the app instructions until reaching the destination. During the experiment, all the participants used their white cane because navigation assistance technologies should aim to complement and not replace the reliable and always functioning traditional travel aids [45]. Two supervisors followed the participant, without interfering with the navigation but being ready to take action in case of potential hazards. After each task, we collected additional subjective feedback as described in Section 4.1.2.

The fourth and last phase of the study collected additional subjective feedback and comments from the participants regarding the whole study and the perceived differences between the two apps, as described in Section 4.1.2.

### 4.5 Participants

The study involved 10 fully blind participants, with no other impairments (see Table 2). This number is in line with prior works in the accessibility field [77, 101] and, based on the conducted power analysis (see Section 4.1.4), sufficient to reach statistically meaningful results. Three participants (**P3**, **P6**, **P10**) had prior experience with the **SOTA** application as they were involved in an experiment focusing on the user interface layer of the system 2 years before the experiment. This was not considered a problem because both applications had the same user interface layer and the study employed a within-group design, comparing how results for each individual varied between the conditions, so no condition was unfairly favored by the prior experience.

Four participants self-identified as female, the others as male. The age of participants was^7^ between 24 and 66 (45 ± 17). Most participants’ (8 of 10) self reported a high (4/5) or very high (5/5) expertise with the use of their mobile device and most (6 of 10) used the mobile device daily for mobility. We also investigated how participants listen to the screen reader during mobility. Four participants reported that they listen to the screen reader while walking using the device speaker (**P1**, **P6**, **P7**, **P8**). **P6** added that she uses stereo earphones depending on the situation. Only **P9** uses stereo earphones to hear spatial audio. The other 5 participants use a single earphone while walking (**P2**, **P3**, **P4**, **P5**, **P10**). All the participants used mobility apps such as Ariadne GPS [49], BlindSquare [20], FourSquare [41], Google Maps [80], iMove [59], Maps [79], Moovit [84], Siri [102], Soundscape [103], VoiceVista [107], Oko [90], Be My Eyes [33], Seeing AI [11] and Envision [32].

Furthermore, we asked the participants how often they travel independently along familiar and unfamiliar routes. Seven participants reported traveling familiar routes daily, the others weekly. Instead, only one participant travels unfamiliar routes daily, the others weekly (one participant), monthly (3 participants) or even more rarely (5 participants).

## 5 EXPERIMENTAL RESULTS

### 5.1 Analysis of Movement Data

All participants completed the tasks and navigated independently during the large part of the experiment. On 26 occasions the supervisor had to temporarily stop the experiment to prevent possible hazards (*e.g.*, although the area was closed to traffic, on a few occasions vehicles passed by) or to recover from major localization problems (*e.g.*, the position markers, which were placed on supports, were moved or knocked over by the wind). In these cases, the supervisor took note of the intervention and later edited the collected data, to exclude the data automatically collected by the system during the supervisor intervention.

**Fig. 6.**
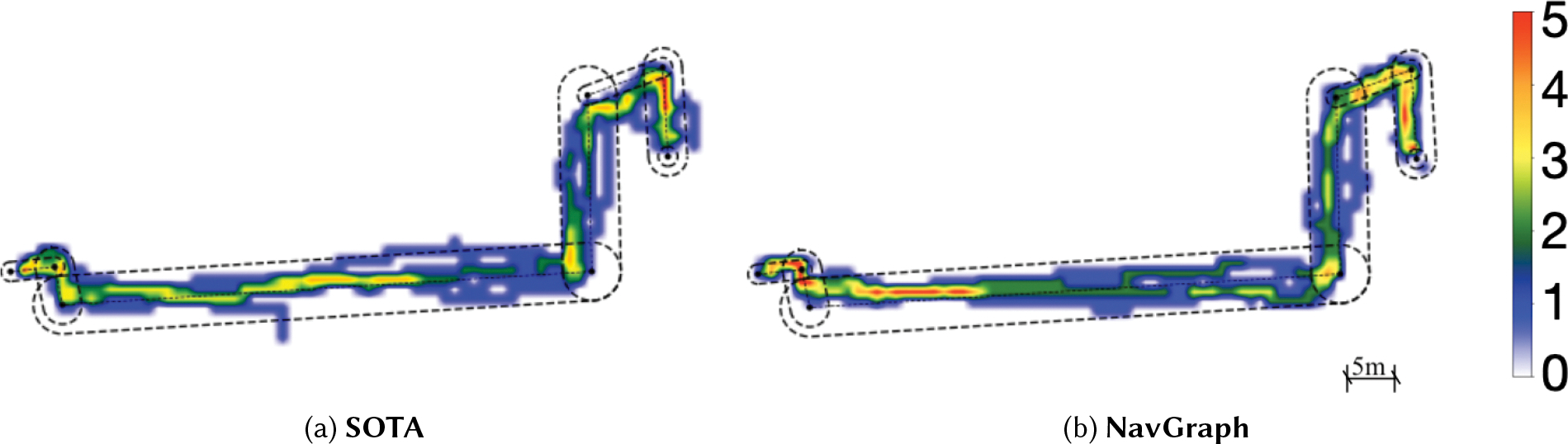
Heatmap of participants’ positions along a route. The scale indicates how many participants traversed each position.

In total, we collected data from 40 paths (4 paths for each participant), equally divided between the two conditions. On average, participants walked for 732.71*s* ± 126.01*s* seconds in total and covered a distance of 414.89*m* ± 14.19*m*. The total number of instructions for each participant was 131 ± 28.37, with 59.4 ± 10.63 rotate instruction, 64.9 ± 16.21 walk instructions and 6.7 ± 3.35 side-step instructions. Fig. 6 shows the heatmap of the paths traveled by the participants along the same route shown in Fig. 5.

#### 5.1.1 Number of Instructions and Duration.

**Fig. 7.**
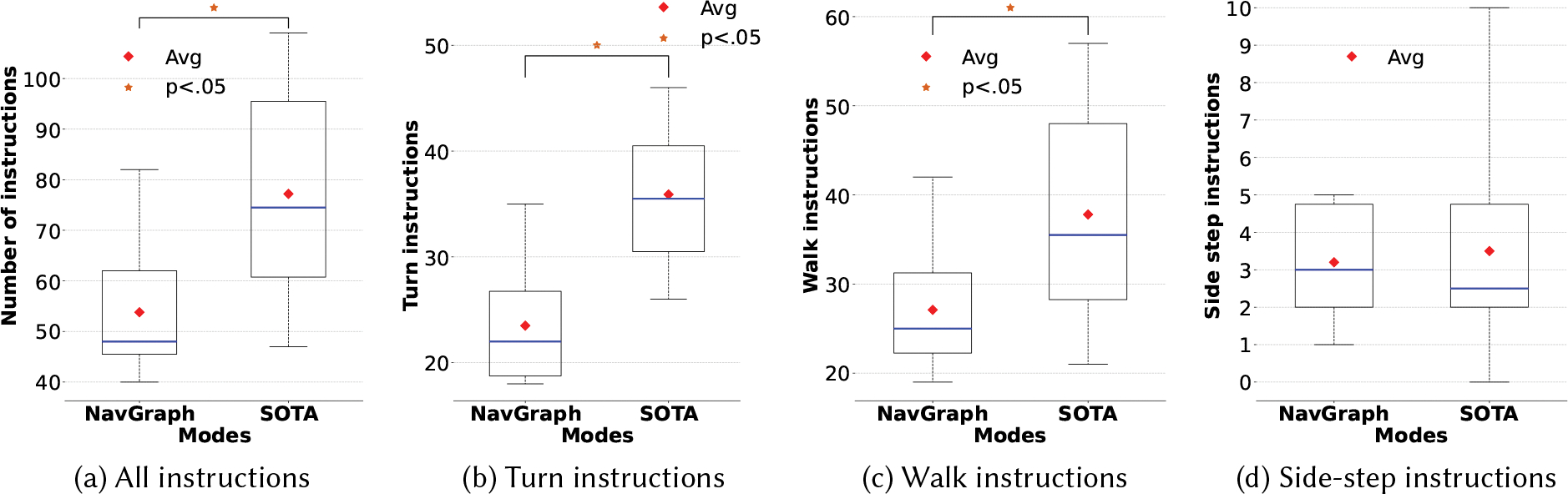
Number of instructions.

Fig. 7 shows a comparison of **NavGraph** and **SOTA** in terms of the number of instructions. Considering the total number of instructions, **NavGraph** (53.8 ± 12.63) had significantly less (p<.01,t(9)=−4.04) instructions than **SOTA** (77.2 ± 19.85). We also conducted additional analyses, considering only turn, walk, or side-step instructions between the two conditions. In this case, both factors (navigation app and instruction type) yielded significant differences (p<.001) as well as their interaction (p<0.05). Pairwise comparisons show that **NavGraph** had significantly fewer turn (**NavGraph**: 23.5 ± 5.45 vs **SOTA**: 35.9 ± 6.8, p<.001,t(9)=−5.97) and walk (**NavGraph**: 27.1 ± 6.7 vs **SOTA**: 37.8 ± 11.63, p<.001,t(9)=−3.25) instructions, while no significant difference was found in the number of side-step instructions (**NavGraph**: 3.2 ± 1.4 vs **SOTA**: 3.5 ± 2.77). Instead, considering the navigation time, no statistically significant difference emerges between (368.32*s* ± 50.39*s*) and **SOTA** (364.39*s* ± 81.85*s*).

#### 5.1.2 Time Outside the Navigation Area.

**Fig. 8.**
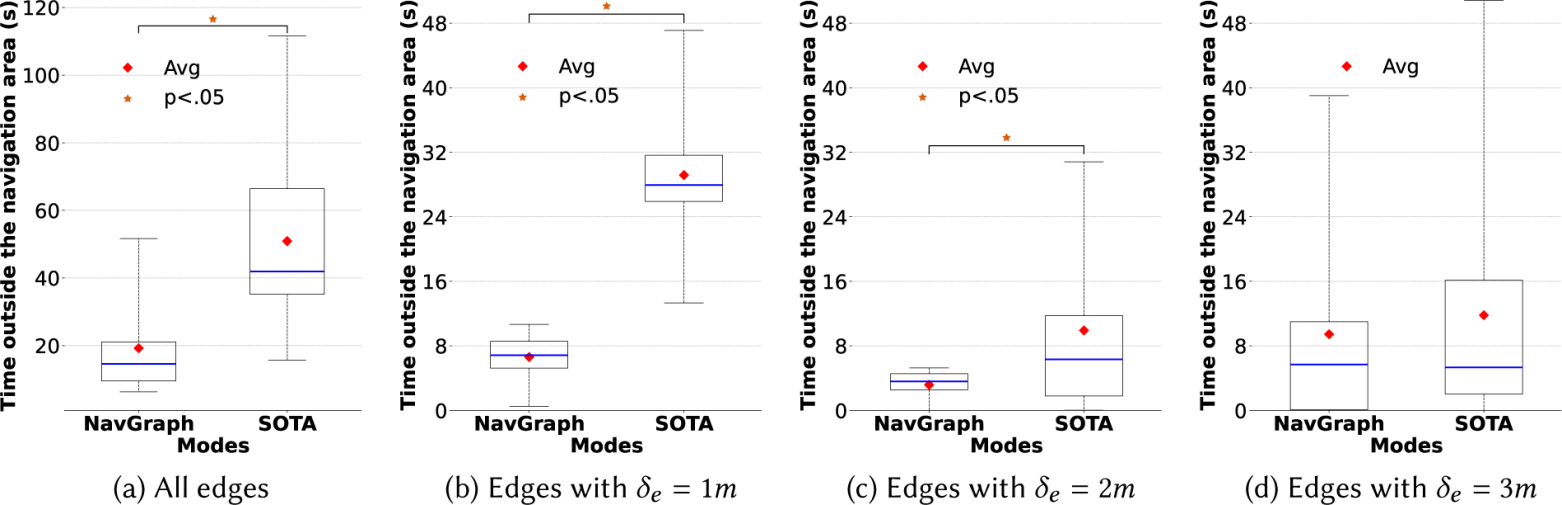
Time outside the navigation area.

Fig. 8 shows how much time the participants spent outside the navigation area (**NavGraph**: 19.23*s* ± 13.55*s*, **SOTA**: 50.88*s* ± 26.32*s*). The difference is statically significant (p<.05,W=0). A related metric is the average distance from the user’s position to the current edge. Also in this case (**NavGraph**: 0.88*m* ± 0.09, **SOTA**: 1.12*m* ± 0.21*m*), the difference is statistically significant (p<.05,t(9)=−3.38).

To better understand the phenomenon, we also analyzed the time spent outside the navigation when the current edge had a navigation area width $\delta_{e}$ equal to 1, 2, and 3 meters (see Figs. 8b, 8c and 8d, respectively). Both factors (navigation app and navigation area width), as well as their interaction, yielded significant differences (p<.001). The difference is more accentuated (**NavGraph**: 6.62*s* ± 2.71*s* vs **SOTA**: 29.71s±8.53s) when $\delta_{e}=1m\;(p<.001,t(9)=-6.86)$ and (**NavGraph**: 3.71*s* ± 1.77*s* vs **SOTA**: 9.91*s* ± 20.59*s*) when $\delta_{e}=2m\;(p<.05,W=5)$. By contrast, when $\delta_{e}=3m$ the difference is not statistically significant.

#### 5.1.3 Instruction Bursts.

A possible undesirable behavior of a navigation system is the occurrence of “instructions bursts”: sequences of requests one after another. Our study of this behavior is motivated partly by [96], which argues that “chunking” navigation instructions into fewer, more meaningful directions helps reduce the user’s cognitive load, compared with turn-by-turn directions that arise often and sometimes unpredictably (including whenever the user’s location or heading deviate too much from the desired path). While **NavGraph** is indeed a form of turn-by-turn directions, the instructions are issued as parsimoniously as possible. As a result, we predict that **NavGraph** leads to fewer bursts of instructions compared with SOTA, which we confirm empirically.

**Fig. 9.**
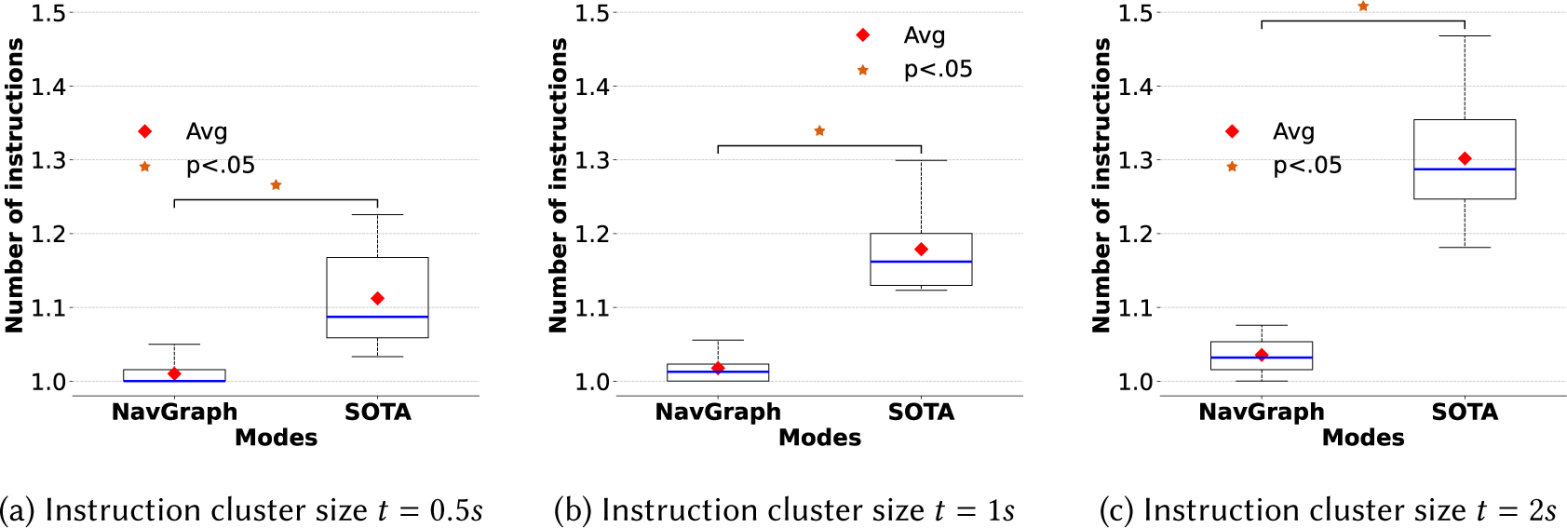
Size of instruction clusters.

To capture this situation, we define the concept of instructions cluster (Section 4.1.1) considering a maximum time distance between two consecutive requests, and we compute the average size of the instructions clusters for each participant, comparing **NavGraph** and **SOTA** (see Fig. 9). For all considered maximum time distances, the differences are statistically significant. Specifically, both the navigation app and maximum time distance factors resulted in significant differences, as well as their interaction (p<.001). For t=0.5s the average size of instruction clusters is 1.01*s* ± 0.02*s* for **NavGraph** and 1.11*s* ± 0.06*s* for **SOTA** (p<0.01,W=0). For t=1s, it is 1.02*s* ± 0.02*s* for **NavGraph** and 1.18*s* ± 0.06*s* for **SOTA** (p<0.001,t(9)=0), and for t=2s it is 1.04*s* ± 0.02*s* for **NavGraph** and 1.30*s* ± 0.08*s* for **SOTA** (p<0.001,t(9)=−9.87).

#### 5.1.4 Learning Effects.

Finally, we have also assessed the presence of learning effects between the first route and the second route for each condition. This analysis was conducted for all the objective metrics (see Section 4.1.1). The obtained results did not highlight any statistically significant difference for any of the considered metrics for this factor in either of the two conditions (**NavGraph** and **SOTA**).

### 5.2 Participants’ Subjective Feedback

**Fig. 10.**
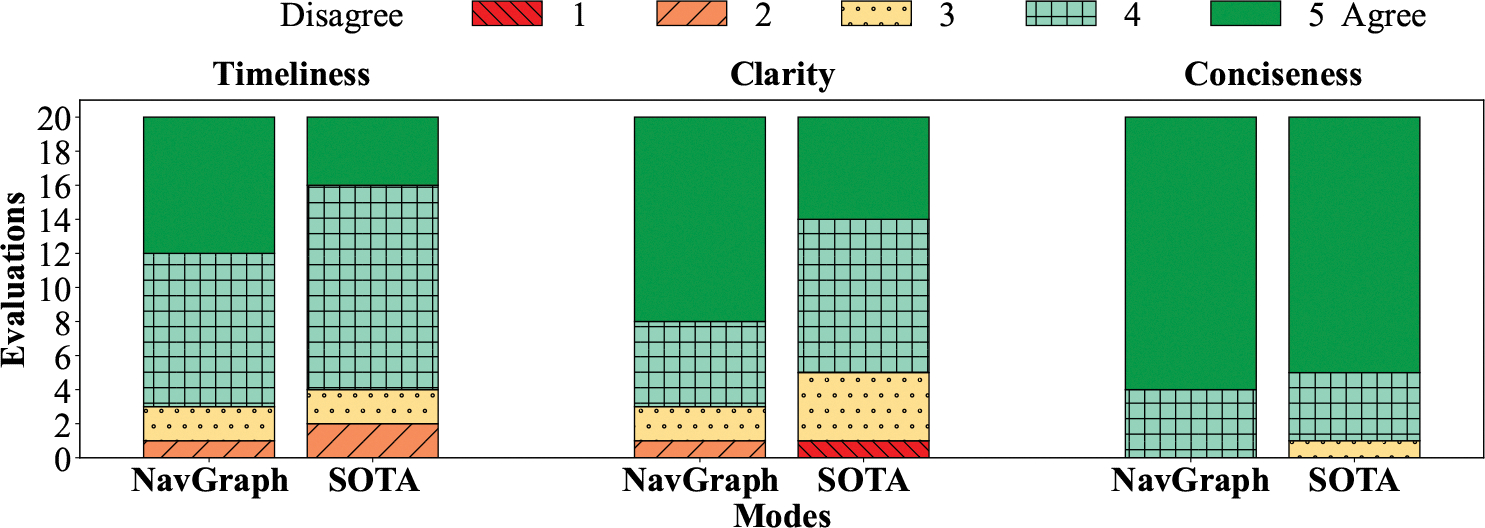
Participant’ subjective feedback for Likert-like items.

Fig. 10 shows the distribution of the answer scores for the Likert-like scale items (see Section 4.1.2). Considering the Clarity metric, **NavGraph** (4.4 ± 0.8^8^) was perceived to be significantly clearer (W=0, p<.05) than **SOTA** (3.95 ± 0.82). Instead, considering **Timeliness** (**NavGraph**: 4.2 ± 0.56, **SOTA**: 3.9 ± 0.7) and **Conciseness** (**NavGraph**: 4.8 ± 0.4, **SOTA**: 4.7 ± 0.56) metrics, no statistically significant differences were found.

Despite the lack of statistically significant differences, for the **Timeliness** metric, half of the participants (**P2**, **P5**, **P8**, **P9** and **P10**) commented that SOTA was too quick in providing instructions.

**P2**: *[With* ***SOTA****] you risk being too prescriptive if you correct me immediately.*

This issue often made the instructions too close to one another, creating *instruction bursts*, which impacted the **Clarity** of the navigation, as reported by five participants (**P2**, **P3**, **P4**, **P5** and **P9**):

**P5**: *[With* ***SOTA****], the instructions kept changing frequently. It was too quick. I couldn’t orient myself properly right away. [With* ***NavGraph****], I was oriented and then I reached the end of a path. I never had to change direction mid-course.*

**P3**: *I received two opposing instructions that were very close to each other, and I didn’t have time to perform the first one; instead, I carried out the second one.*

Similarly, **P4** also observed that, with **SOTA** in two cases he had to immediately correct himself after the first instruction, such as turning left and then right.

Considering **Completeness**, 4 participants (**P3**, **P6**, **P7**, **P9**) observed that both navigation apps lack a functionality to identify and avoid objects. Instead, considering the **Conciseness** aspect, no additional comments were provided.

In the final questionnaire, the answers to the **Quantity of instructions** question show that four participants (**P5**, **P7**, **P9**, **P10**) perceived that **SOTA** provides more frequent instructions than **NavGraph**, while two (**P2**, **P8**) perceived the opposite, and four did not perceive any difference (**P1**, **P3**, **P4**, **P6**). Finally, considering the General differences question, **P1** and **P2** perceived the difference in the lateral step instruction, highlighting that only one step was required in **SOTA** while **NavGraph** provided a stronger correction, and **P5** noted that sometimes **SOTA** would provide instructions even when the previous one was not completed.

## 6 DISCUSSION

We discuss the key results of our investigation, namely the benefits of the *Parsimonious Instructions* design principle that we adopt in our solution (Section 6.1), and the benefits of the problem decomposition approach (Section 6.2). We also address the main limitations of our methodological approach (Section 6.3).

### 6.1 Benefits of the *Parsimonious Instructions* Design Principle

#### 6.1.1 Advancement With Respect to the State of the Art.

The experimental results provide concrete evidence that the *Parsimonious Instructions* design principle, implemented in the *Routing* and *Guidance* modules of the **NavGraph** system, has a significant impact on the navigation assistance and on the resulting user experience perceived during the assisted navigation. Specifically, our experiments show that the adoption of the *Parsimonious Instructions* design principle can reduce a) the number of instructions, b) instructions bursts, and c) the time spent outside the navigation area.

Prior works mention the importance of reducing the cognitive load inside navigation assistance solutions for BLV people in order to make the system less invasive and more secure, thereby allowing the user to maintain awareness of their surroundings [6, 91, 106, 108]. However some of the prior works only provide ad-hoc solutions for specific contexts and do not evaluate their impact. For example, in some cases, instructions are reduced by limiting the types of the conveyed information or providing them on demand only [91, 97]. In our work, instead, we state the *Parsimonious Instructions* design principle, we implement it through three policies, and we evaluate its impact on the navigation safety and user experience.

As shown in Section 5.1.1, the application of the *Parsimonious Instructions* design principle results in a reduced number of instructions compared to a state of the art solution. Reducing the number of instructions also impacts the instructions bursts (see Section 5.1.3). The instructions bursts, as reported in prior literature [8, 50, 96] and as also highlighted by our participants (see Section 5.2), have a negative impact on the clarity of a navigation system. Indeed, **NavGraph** system was also perceived to be significantly clearer than **SOTA**.

Finally, reducing the time spent outside the navigation area is also relevant: the navigation area represents a location where the user is expected to be able to move safely (*e.g.*, the sidewalk), while the region outside the navigation area (*e.g.*, the roadway) can be potentially hazardous. This is particularly important when safe areas are narrow (*e.g.*, pedestrian crossings), or not outlined with strong physical cues (*e.g.*, curbs), and the pedestrian can leave the safe area without noticing it [18, 31, 54]. Indeed, **NavGraph** displayed a significant reduction of the time spent outside the navigation area, in particular for paths that have a narrower safe area (see Section 5.1.2).

#### 6.1.2 *Applications of the* Parsimonious Instructions *Design Principle in Navigation Applications.*

The *Parsimonious Instructions* design principle, defined in this paper, is implemented inside **NavGraph**, a navigation assistance prototype app, through the following three policies.

1. A routing algorithm that solves the **Target update** problem without requiring the user to get in close proximity of the current target.
2. A guidance policy that adopts “strong” instructions that guide the user into a position (or orientation) where the same instruction is less likely to be provided. This is in contrast with “weak” instructions that guide the user to do a movement that temporarily corrects the user’s position (or orientation) but can potentially require a repetition of the same instruction soon afterwards.
3. A guidance policy that is more tolerant of the user orientation: as long as the user is approximately heading towards the current target area (not necessarily the current target), their orientation is not corrected.

**NavGraph** implements these policies by extending a prototype previously proposed in the literature [2] but the same policies can be adopted in other existing applications, including both research prototypes and commercial system.

The application of the first and third policies listed above requires defining the concept of navigation area in the considered space representation. Although this is conceptually simple, practical problems could arise with the specification of the data structure. For example, when large areas need to be mapped, the procedure can be time-consuming and error prone [46]. We foresee two possible solutions to address, or at least mitigate, this problem. First, automatic mapping tools can be used to automatically (or semi-automatically) create the space representation using visuo-inertial techniques [87]. Second, augmented-reality applications can be designed to visually represent the space representation. Such an application would serve as a tool to support a sighted person in the debugging of the data structure and possibly in its creation as well.

The application of a “strong” instructions policy can be easily implemented in existing systems. **NavGraph** implements this policy through the concept of restricted navigation area. Note that, differently from the navigation area, the restricted navigation area does not represent physical constraints in the environment (*e.g.*, a corridor width) and it does not add complexity in the definition of the data structure because it can be automatically computed from the navigation area. In our experiments we used a restricted navigation area that is half as wide as the navigation area. We believe that for most applications it could also be effective to define a restricted navigation area with fixed width.

### 6.2 Benefits of the Problem Decomposition Approach

In this work we decompose the overall problem of navigation assistance for people who are BLV into four sub-problems: localization, routing, guidance, and user interface (see Section 2.1). This approach extends the one adopted in the broader literature on navigation systems (not specifically for BLV travelers) where a subdivision into three layers (localization, routing, user interface) was proposed [65].

The guidance layer addresses the problem of selecting the instructions to guide the user toward the current target. This problem exists for general purpose navigation systems (not specifically designed for BLV people) as well, and is addressed as a part of the user interface layer. However, we argue that this problem is particularly relevant, and hence worth addressing in a separate layer, for the navigation of BLV people, due to the need to safely guide them toward the destination without overwhelming them with too much information [2, 73, 81, 91, 98, 105].

Explicitly decomposing the navigation problem into separate sub-problems (or layers) has two main advantages. First, it is a form of problem conceptualization that can be used to analyze existing solutions and classify them depending on the sub-problem(s) they address (see Section 2). Second, the identification of four independent software components facilitates software re-use. For example, the app prototype that we developed for the experiments can be easily modified to adopt a different *Localization* module (*e.g.*, based on Bluetooth beacons as in the Navcog system [97]). It is also possible to experiment with different *User Interface* modules as well.

### 6.3 Limitations

We discuss three key limitations of our work: due to the experimental setting (Section 6.3.1), due to the apparatus used (Section 6.3.2), and due to the limited demographic and cultural diversity among the participants (Section 6.3.3).

#### 6.3.1 Experimental Setting Limitations.

In this paper we evaluated the impact of the proposed navigation approach in a real-world scenario. Specifically, the experimental evaluation was conducted on a university campus for three main reasons: to limit the hazards for the participants, to limit unpredictable obstacles along the route (*e.g.*, a parked bicycle) that could impact the study results, and to test the system in an environment where participants would not be guided by environmental features (*e.g.*, a corridor) that can be used as references, haptically (*e.g.*, by following a corridor wall with the white cane) or acoustically (thanks to echolocation, BLV travelers can, for example, sense the presence of an open door in a corridor).

These characteristics, however, might not be present in other scenarios, and hence could influence the navigation process and its outcome. For example, in our study, the participants knew that, in case of a hazard, the supervisor would intervene. This might have impacted the way the participants navigated or affected their walking speed. Similarly, in an environment with unpredictable obstacles, the participants would have most likely had to avoid them, which would have influenced their walking routes. Also, while there was some noise in the area due to the presence of numerous students and a few passing cars, the overall background noise was different than in a typical outdoor environment with many passing vehicles. The difference in the background noise could have potentially influenced the ability of the users to perceive the instructions and the sonifications.

There are also two factors that made the experimental setting harder to navigate than routes usually traversed by the participants. First, the participants had no previous knowledge of the experimental routes. This is particularly challenging because BLV individuals most commonly travel on familiar routes or study the route beforehand, as already observed in the literature [56] and also reported by the participants in our experiment (see Section 4.5). Second, the participants had no environment reference points, and in particular no haptic references. One common practice among BLV individuals who use the white cane (as all our participants did) is to follow haptic references, like walls or sidewalk edges. In the experimental setting, we intentionally decided to avoid these references, so that they would not affect the results, but clearly, this makes the walking task more challenging.

#### 6.3.2 Apparatus Limitations.

Study results may have also been influenced by the experimental apparatus, for three reasons. First, we did not measure the impact of the localization accuracy since this was out of the scope of the investigation. However, since the navigation instructions depend on the system localization accuracy, we acknowledge that this factor could be relevant as well. To account for this issue, we positioned the localization markers approximately every 5m, thus mitigating the localization drift. We empirically observed that the drift effect was limited with the only exception (excluded in the analysis of the results) in which the location markers were accidentally moved or knocked down (*e.g.*, by the wind).

Second, as described in Section 4.2, we designed the evaluation to compare **NavGraph** with another prototype (**SOTA**) and not with a commercial application. While this was necessary to evaluate the impact of the *Parsimonious Instructions* design principle on the navigation assistance of people who are BLV, we acknowledge that future comparisons with existing solutions are needed to ensure that the proposed approach is sound and generalizable.

Third, another peculiarity of the study is the use of a chest harness. This study design choice helped to simplify the experimental protocol because, if the participants held the device with their hands, it would have been harder to distinguish body rotations from hand rotations. Participants would have also needed to concentrate on pointing the device at the right angle (*e.g.*, not towards the ground), which is known to be hard for BLV individuals [82]. However, a chest harness may not be a practical solution for a number of reasons, including that it may be deemed cumbersome by users and the smartphone can be easily stolen from it. Alternative solutions could be wearable devices, like smart glasses, and other types of bags or ways of securing the smartphone.

#### 6.3.3 Demographic and Cultural Bias.

In this work, since the experimental setting was constructed in a specific real-world environment, we could only recruit participants from the same geographical area. Furthermore, in order to limit confounding factors that could impact the results, we only recruited participants who are fully blind. As a result, the study involved a small number of participants, a limitation that is common in the field of research on assistive technologies [101].

## 7 CONCLUSION AND FUTURE WORK

In this paper, we introduced the *Parsimonious Instructions* design principle, which aims to reduce the number of instructions while providing safe navigation guidance to BLV pedestrians. Grounded in this design principle, we developed **NavGraph**, a modular navigation system tailored for BLV travelers. The system is organized into four modular layers, enabling clear division of responsibilities. Our user study shows that **NavGraph** reduces the number of instructions and minimizes time spent outside safe navigation zones, while also improving clarity. These findings demonstrate the impact of the proposed design principle.

This paper impacts future research in four main directions. First, it provides empirical evidence that reducing cognitive load during navigation has a significant impact on safety and user experience. Therefore, it will motivate the reduction of the cognitive load, a relevant objective in the analysis of future navigation systems. Second, this paper presents the *Parsimonious Instructions* design principle that can be adopted as a general principle in the design of future navigation systems for BLV people. Similarly, this paper presents three policies to implement this design principle that can be adapted to most navigation systems. Third, this paper motivates the importance of separating the navigation problem into four sub-problems, thus highlighting the need to address the four problems individually and, in particular, to explicitly consider the *guidance* sub-problem which we demonstrate is particularly relevant for BLV navigation assistance. Fourth, the methodology adopted in this paper can be reproduced by experimenting with other navigation systems. In particular, it will be possible for researchers to modify an existing navigation system (a commercial application or a research prototype) adopting the *Parsimonious Instructions* design principle, and empirically evaluating the effects of the application of this principle.

This work opens up several research directions. First, **NavGraph** can be evaluated in multiple environments, both indoor (*e.g.*, museums or airports) and outdoor (*e.g.*, roads with loud traffic noise). This extension would help to understand whether the proposed approach generalizes to diverse environments and contexts. Second, **NavGraph** can serve as a technological platform to support the design and evaluation of novel solutions across each of the four identified layers. For instance, future work could explore alternative routing strategies and compare their effect on the user experience by changing the routing layer only. Similarly, other positioning techniques (*e.g.*, based on the LIDAR sensor) could be integrated in **NavGraph** and compared. Another possible extension involves enhancing **NavGraph** functions, such as by integrating obstacle detection and avoidance features. In this context, it would be interesting to investigate how the principle of *Parsimonious Instructions* applies to these new functionalities. An additional research direction is the application of the *Parsimonious Instructions* design principle to the navigation support of users with low vision who rely on residual sight. This raises the research question of whether the *Parsimonious Instructions* design principle is also beneficial for visual information and, if so, how graphical and auditory interfaces should be designed to embody this principle.

## Supplementary Material

MP4 File - Differences between NavGraph and state-of-the-art

## Figures and Tables

**Table 1.** Differences between **NavGraph** and **SOTA**

Module	NavGraph	SOTA
**Routing**	Updates current target when the user enters in the navigation area of the next edge.	Updates current target when the user is in the proximity of the current target
**Guidance**	When issuing side-step instructions, guides the user to the restricted navigation area.	Single side-step instructions
**Guidance**	Corrects user’s orientation if the user is not directed toward the intersection of the navigation areas of current and next edges (as in Fig. 4).	Corrects user’s orientation if misaligned by more than 30° with respect to the current target
**User Interface**	Side-step instruction followed by sonification.	Side-step instruction not followed by sonification.

**Table 2.** Participants’ demographic information.

ID	Age	Gender	Impairment		Mobile		Screen reader		Route		SOTA
			Level	Onset	Expertise	frequency	Speed	Voice	Familiar	Unfamiliar	Expertise
P1	55	M	Fully Blind	birth	4	Daily	70	M	Weekly	Monthly	No
P2	42	F	Fully Blind	birth	4	Daily	80	F	Daily	Daily	No
P3	53	F	Fully Blind	birth	5	Weekly	75	M	Daily	Rarely	Yes
P4	46	M	Fully Blind	birth	5	Daily	>=75	F	Daily	Weekly	No
P5	24	M	Fully Blind	birth	5	Daily	65	F	Daily	Rarely	No
P6	28	F	Fully Blind	birth	5	Weekly	70	M	Weekly	Monthly	Yes
P7	66	M	Fully Blind	age 6	3	Daily	55	M	Daily	Rarely	No
P8	62	M	Fully Blind	age 10	5	Weekly	65–85	F	Daily	Monthly	No
P9	26	F	Fully Blind	birth	5	Weekly	60	M	Weekly	Rarely	No
P10	25	M	Fully Blind	birth	3	Daily	95	F	Daily	Rarely	Yes

## Footnotes

1 Global Navigation Satellite System is an umbrella term that encompasses different global satellite localization systems, currently: the US-based GPS, the European Galileo, the Russian GLONASS and the Chinese BDS.

2 Planning, conduct, and reporting of the work are consistent with the general principles enunciated in [15].

3 The experiments were conducted in Italian; we report the English translation.

4 Source code of **NavGraph** is available at: https://anonymous.4open.science/r/NavGraph-6C86

5 Source code of **SOTA** is available at:https://anonymous.4open.science/r/SOTA-E029

6
https://developer.apple.com/augmented-reality/arkit/

7 We use the notation x±y to denote average x and standard deviation y.

8 We report means for multipoint items as they were found to better indicate central tendency than medians [74].
